# Diagnosis of hepatocellular adenoma in men before onset of diabetes in HNF1A‐MODY: Watch out for winkers

**DOI:** 10.1111/liv.14235

**Published:** 2019-09-13

**Authors:** Martijn P. D. Haring, Titia M. Vriesendorp, Jolien S. Klein Wassink‐Ruiter, Robbert J. de Haas, Annette S. H. Gouw, Vincent E. de Meijer

**Affiliations:** ^1^ Department of Surgery University Medical Center Groningen University of Groningen Groningen the Netherlands; ^2^ Department of Internal Medicine Isala Diabetes Center Zwolle the Netherlands; ^3^ Department of Genetics University Medical Center Groningen University of Groningen Groningen the Netherlands; ^4^ Department of Radiology University Medical Center Groningen University of Groningen Groningen the Netherlands; ^5^ Department of Pathology and Medical Biology University Medical Center Groningen University of Groningen Groningen the Netherlands

**Keywords:** hepatocellular adenoma, HNF1A‐MODY, liver adenomatosis, treatment algorithm

## Abstract

Hepatocyte nuclear factor 1A (*HNF1A*) maturity‐onset diabetes of the young (MODY) is a monogenetic, autosomal dominantly inherited form of diabetes. HNF1A‐MODY is associated with *HNF1A*‐inactivated hepatocellular adenoma (H‐HCA) formation. Hepatocellular adenoma (HCA) are benign liver tumours and related complications are rare but serious: hepatic haemorrhage and malignant transformation. Guidelines recommend resection of all HCA in men and do not take any co‐occurring metabolic disorders into account. We report a family with HCA preceding diabetes mellitus. Male index patient presented with numerous, irresectable HCA. After initial diagnostic and aetiologic uncertainty *HNF1A* germline mutation *c.815G>A (p.Arg272His)* was confirmed 8 years later. No HCA‐related complications occurred. His diabetic mother was diagnosed with HCA after severe hepatic haemorrhage years before. HNF1A‐MODY should be considered in (non‐)diabetic (male) patients with H‐HCA. We advocate liver biopsy and, if necessary, genetic analysis to precede any intervention for HCA in males and screening for HCA in HNF1A‐MODY patients.

**Abbreviations**
DM: diabetes mellitus
HCA: hepatocellular adenoma
H‐HCA: HNF1A‐inactivated HCA
HNF1A: hepatocyte nuclear factor 1A
LA: liver adenomatosis
MODY: maturity‐onset diabetes of the young
MRI: magnetic resonance imaging

## 1 INTRODUCTION

Maturity‐onset diabetes of the young (MODY) is a monogenic, autosomal dominant, non‐ketotic form of diabetes mellitus (DM), representing genetic, metabolic and clinical heterogeneity. Because of a heterozygous germline mutation of the hepatocyte nuclear factor 1A (*HNF1A*) gene, HNF1A‐MODY (formerly MODY3) is the most common subtype. HNF1A‐MODY is associated with diabetes onset before the age of 25 years and gestational diabetes, and is treated best with sulfonylurea derivatives.^1^, ^2^, ^3^

Transcription factor *HNF1A* interacts with DNA as a homodimer or heterodimer with *HNF1B* to regulate cellular function, including carbohydrate metabolism, lipid transport and detoxication, and is present in various organs of endodermal origin.^1^ *HNF1A* is also an autosomal dominant inherited tumour suppressor gene. Somatic bi‐allelic mutations of *HNF1A* are associated with hepatocellular adenoma (HCA) formation.^4^, ^5^ HCA are rare, benign liver tumours at risk for haemorrhage (15%) or malignant transformation to hepatocellular carcinoma (HCC, 5%).^5^ HNF1A‐inactivated HCA (H‐HCA) are usually sporadic cases, which form 35% of HCA.^4^, ^5^ About a third of patients with H‐HCA carry a *HNF1A* germline mutation in one allele, thereby causing H‐HCA formation following a second, somatic mutation in the wild‐type *HNF1A* allele.^4^ Liver adenomatosis (LA) has first been described in the 1960s and has later been defined by the occurrence of 10 or more HCA.^6^, ^7^ It is a different entity from regular HCA, as it occurs more frequently in the presence of underlying liver disease such as HNF1A‐MODY or glycogen storage disease.^8^, ^9^, ^10^ The 2016 European guideline on benign liver tumours acknowledges the formation of HCA in metabolic disorders such as HNF1A‐MODY, and recommends HCA resection in males, irrespective of size or concurring metabolic diagnosis.^11^ Phenotype penetrance varies in families with identical *HNF1A* mutations, although MODY is typically diagnosed prior to H‐HCA.^12^

## 2 MATERIALS AND METHODS

We report a family with a *HNF1A* germline mutation and high penetrance of H‐HCA. Information was obtained from the electronic patient file after all patients gave consent. Immunohistochemistry was performed to analyse tissue samples for expression of c‐reactive protein (CRP), serum amyloid A (SAA), liver fatty acid binding protein (LFABP) and glutamine synthetase (GS). Genetic analysis after leucocyte DNA extraction was performed by next‐generation sequencing of the MODY gene panel. The panel involves 13 MODY genes: *HNF4A, GCK, HNF1A, HNF1B, PDX1, NEUROD1, KLF11, CEL, PAX4, INS, BLK, ABCC8, KCNJ11* and included multiplex ligation‐dependent probe amplification. Follow‐up for all family members by magnetic resonance imaging (MRI) was at least 2 years.

## 3 RESULTS

**Figure 1:**
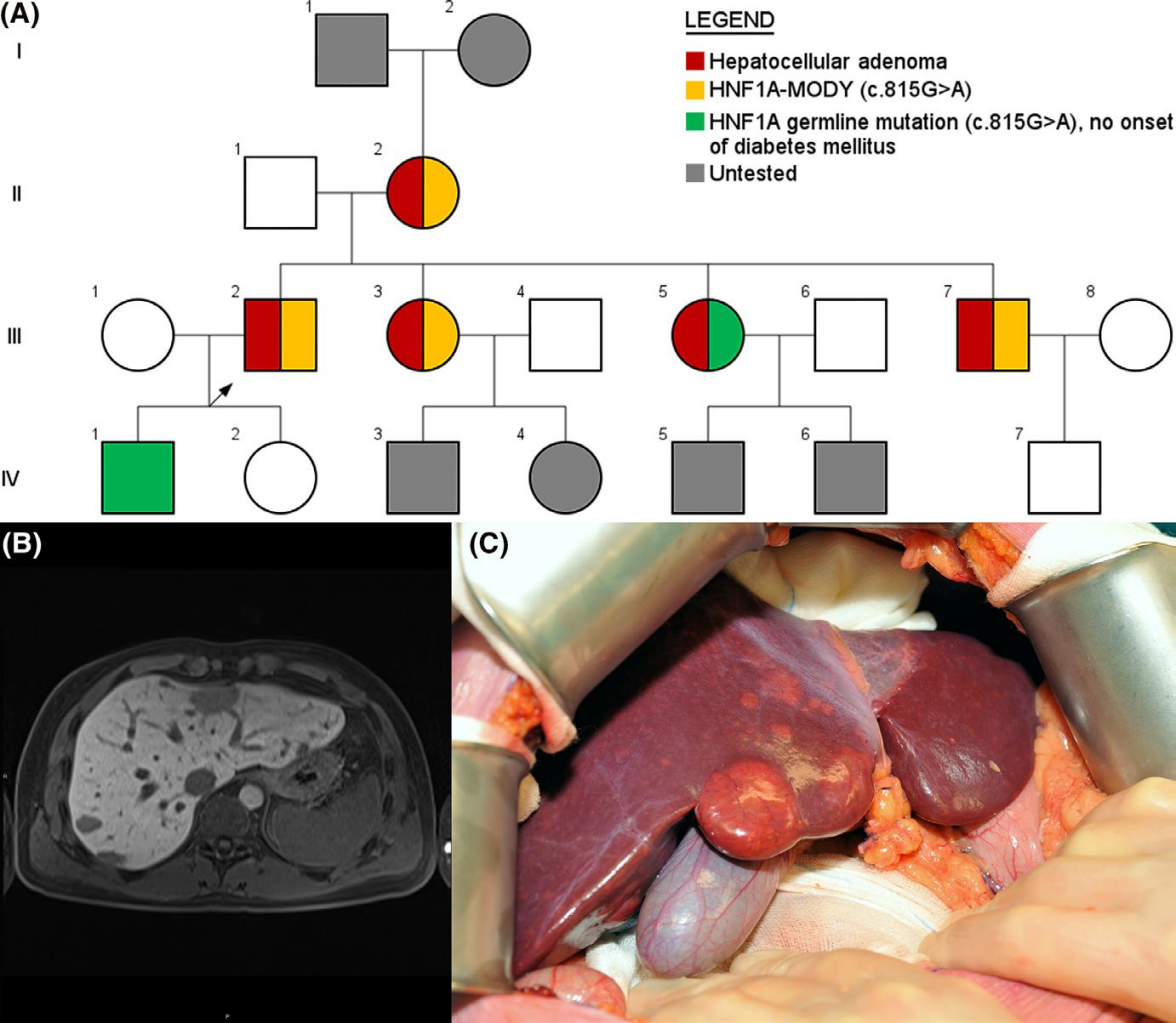
A, Pedigree of the family. A patient identification number is formed by Roman numeral (generation), followed by Arabic number (individual within generation). Index patient (III:3) is denoted with an arrow. B, Pre‐operative axial T1‐weighted fat‐saturated, gadoxetic acid‐enhanced MRI of index patient demonstrating numerous, bi‐lobar liver tumours. C, Intra‐operative macroscopic appearance of index patient's liver showing a diffuse bi‐lobar spread of liver tumours. Legend: red, hepatocellular adenoma diagnosis; green *HNF1A c.815G>A (p.Arg272His)* mutation, but no onset of diabetes; yellow *HNF1A c.815G>A (p.Arg272His)* mutation; grey, no genetic analysis performed. *HNF1A*, hepatocyte nuclear factor 1A; MRI, magnetic resonance imaging

A 25‐year‐old man (Figure 1A, III:2, index patient) without a medical history was presented with right hypochondriac ache. Ultrasonography revealed two liver tumours, leading to referral to our centre. Serum alpha‐foetoprotein, carcinoembryonic antigen and cancer antigen 19‐9 levels were normal. Computed tomography (CT) revealed numerous, bi‐lobar liver lesions with arterial enhancement and washout. Contrast‐enhanced MRI could not differentiate between benignancy and malignancy (Figure 1B). Histological and immunohistochemical analysis after percutaneous liver biopsy diagnosed HCA, but could not exclude well‐differentiated HCC.

During laparotomy, an extensive, liver‐wide spread of tumours, ranging in size from microscopic up to 40 mm was observed—preventing any tumour‐free hepatic remnant (Figure 1C). Histology of the resected specimen showed HCA without malignant features. Immunohistology showed normal expression pattern of CRP, SAA and GS but loss of LFABP—pathognomonic for H‐HCA. Management by wait‐and‐see was decided upon. When DM was diagnosed 8 years later, type 1 was considered less likely because of negative anti‐islet cell antibodies‐2 and antiglutamic acid decarboxylase tests.

One year before, index patient's sister (Figure 1A, III:3) was presented with gestational diabetes. MODY was suspected because hyperglycaemia persisted post‐partum. Genetic analysis after leucocyte DNA extraction confirmed a germline mutation in exon 4, *c.815G>A* (*p.Arg272His),* in the *HNF1A* gene DNA‐binding homeodomain, confirming HNF1A‐MODY. Hepatobiliary contrast‐enhanced MRI visualized three small liver tumours, highly suspected for HCA. This germline mutation and HCA diagnosis led to aetiologic reconsideration of the HCA and DM in the index patient. Subsequent genetic analysis of the index patient confirmed the same germline mutation in the *HNF1A* gene. She had used an estrogen‐containing oral contraceptive pill for 18 years at the time of diagnosis.

Thereafter, all first‐ and second‐degree family members were screened for mutations in the *HNF1A* gene (Figure 1A). The mutation was maternally inherited (Figure 1A, 2:II). Index patient's mother was diagnosed with DM at the age of 21. Aged 42 years, she underwent emergency laparotomy after spontaneous hepatic haemorrhage. Histopathological analysis of intra‐operative liver biopsies showed necrosis and steatotic hepatocytes. HCA was suspected, but unconfirmed. Post‐operative CT revealed multiple additional arterially enhancing liver lesions with a maximum size of 43 mm. Most recent hepatobiliary contrast‐enhanced MRI, at the age of 61, showed regression of the largest tumour to 8 mm, most likely HCA. Onset of the menopause was around her 50th year.

Index patient's brother (Figure 1A, III:7) was 26 years when diagnosed with DM, and genetic analysis confirmed the same germline *HNF1A* mutation. MRI with a hepatobiliary contrast agent showed three steatotic HCA of 9‐13 mm, stable in size during follow‐up. The same germline *HNF1A* mutation was also confirmed for index patient's other sister (Figure 1A, III:5). To date, she does not have symptoms of DM. Hepatobiliary contrast‐enhanced MRI of the liver revealed several tumours (maximum 10 mm), most likely HCA, which remained stable during follow‐up.

## 4 DISCUSSION

Our report describes a family with a *HNF1A* germline mutation and a high penetrance of HCA, varying from single HCA to LA. Interestingly, both the index patient and also likely one of his sisters presented with HCA prior to DM. In one family member, index patient's mother, severe hepatic haemorrhage led to the diagnosis of HCA.

Haddouche et al have described the largest series of patients with a *HNF1A* germline mutation.^10^ They report 87.5% of their cases demonstrating both DM and LA.^10^ In seven of 30 HNF1A‐MODY patients without DM, LA was revealed by screening. In addition, they report three patients who were diagnosed with HCA only after severe hepatic bleeding.^10^ Barbier et al observed four cases of LA prior to DM in a family with a *HNF1A* germline mutation.^9^ In two male patients, HCA was diagnosed by haemorrhage.^9^

Malignant transformation from HCA to HCC is mostly observed in HCA carrying either an exon 3 b‐catenin mutation, or in HCA occurring in males.^5^, ^13^ H‐HCA is less known for this outcome, but should not be neglected completely. About 1.5% of all HCC carry a *HNF1A* mutation, and a HNF1A‐MODY family with H‐HCA‐induced primary hepatic malignancies has been reported.^14^, ^15^

The expression of the diabetic phenotype of HNF1A‐MODY is regulated by modifier genes.^16^ Similar regulation by a currently unknown modifier gene could explain observations of LA prior to or after onset of diabetic symptoms. Our observed *HNF1A* germline mutation in the DNA‐binding homeodomain (amino acids 198‐281) in exon 4, *c.815G>A (p.Arg272His)* has previously been described.^17^ The location of the mutation in the DNA‐binding homeodomain could explain the high penetrance of HCA in the family described in this report.^12^

HNF1A‐MODY should always be taken into consideration when H‐HCA are diagnosed, even without concurring DM and especially if mutations in the DNA‐binding domain of the *HNF1A* gene are found. We advocate pre‐operative percutaneous liver biopsy and immunohistochemical and genetic analysis of both liver tissue and peripheral blood for *HNF1A* mutations if multiple steatotic HCA are diagnosed in male and female (diabetic) patients. Subsequent wait‐and‐see policy should be guided by alpha‐foetoprotein and adequate imaging.

Diagnosing HNF1A‐MODY accordingly may prevent H‐HCA‐related complications, and allow for correct diabetes treatment for both the patient and his or her family. Even without clinical signs of DM, ruling out HNF1A‐MODY may be relevant especially in men with H‐HCA. For men diagnosed with concurring HNF1A‐MODY, close monitoring might be more appropriate. Although the (bleeding) risks of their HCA should not be underestimated, the current dogma to always intervene with surgical intervention should be reconsidered.

## CONFLICT OF INTEREST

None of the authors declared conflict of interest.

## AUTHOR CONTRIBUTIONS

All authors contributed to the drafting and revision of the manuscript. They interpreted the clinical information from their specialty and supervised the correct interpretation of results.
